# Flow cytometry based platelet activation markers and state of inflammation among subjects with type 2 diabetes with and without depression

**DOI:** 10.1038/s41598-022-13037-z

**Published:** 2022-06-16

**Authors:** Shyamkrishnan R, Gautom Kumar Saharia, Suravi Patra, Debapriya Bandyopadhyay, Binod Kumar Patro

**Affiliations:** 1 Department of Biochemistry, AIIMS, Room no. 134, Academic Block, Bhubaneswar, Odisha 751019 India; 2 Department of Psychiatry, AIIMS, Bhubaneswar, Odisha 751019 India; 3 Department of Community Medicine and Family Medicine, AIIMS, Bhubaneswar, Odisha 751019 India

**Keywords:** Biochemistry, Neuroscience

## Abstract

Type 2 Diabetes Mellitus (T2DM) and Major Depressive Disorder (MDD) are highly disabling disorders associated with a multitude of vascular complications. Platelets are known to play a role in the pathogenesis of vascular complications in both T2DM and MDD. These complications could increase in patients with comorbid diabetes and depression. To quantify and compare flow cytometry based platelet activation markers and the inflammatory state between individuals of diabetes with depression, individuals of diabetes without depression and healthy controls. Out of 114 participants, each study group contained 38 participants in diabetic group, diabetics with depression group and matched control group. Diabetes was diagnosed with the American Diabetes Association (ADA) criteria. Screening of MDD was done with Patient Health Questionnaire 2 (PHQ2) and severity of depression assessed with Hamilton Depression Rating (HAM-D) scale. Platelet markers CD41, CD42b, CD62P and CD63 were assayed using flow cytometer. Platelet count, surface expression of platelet activation markers CD62P and CD63, hs-CRP, insulin and HOMA-IR score differed significantly between the groups. Post hoc analysis showed significantly high CD63 expression in patients with comorbid diabetes and depression compared to those having diabetes without depression. Patients with comorbid diabetes and depression have enhanced platelet hyperactivation and a pro inflammatory state which increases susceptibility to vascular complications.

## Introduction

Type 2 Diabetes Mellitus (T2DM) is defined by the World Health Organization as a metabolic disorder of multiple etiology characterized by chronic hyperglycemia with disturbances of carbohydrate, fat and protein metabolism resulting from defects in insulin secretion, insulin action, or both. It is estimated to have a prevalence as high as 8.3% globally, which is expected to rise up to 9.5% by 2030. India is expected to have the highest number of patients with diabetes in the world by that year^1^.

Diabetes Mellitus leads to both microvascular and macrovascular complications. Diabetic neuropathy, retinopathy and nephropathy are the major microvascular complications seen in patients with diabetes^2^. Increased incidence of coronary artery disease (CAD), peripheral vascular disease (PVD) and cerebrovascular disease (CVD) resulting from accelerated atherosclerosis are the macrovascular complications. Up to 80% mortality of diabetes is associated with thrombosis and related complications^3^. A key role is played by platelets in the pathophysiology of these complications. Multiple signalling pathways in platelets are activated in a diabetic milieu resulting in long standing mortality and morbidity^4^.

Major Depressive Disorder (MDD) is a disease attributed to multifactorial etiology including psychological, social, biological risks and associated stressful events. It is the second major cause of disability among individuals of 15–44 years’ age group^5^. Increased risk of cardiovascular diseases is seen in patients with MDD. Platelet hyperactivity and endothelial dysfunction are the major etiological factors that link MDD and CVD. Various studies have elucidated the complex mechanisms by which platelets are activated and endothelium is damaged in subjects with MDD^6^ as well as the association of classical platelet parameters with CRP in persons with specific depressive symptoms and cognitive impairment factors^7^.

Both T2DM and MDD are highly disabling diseases. A bidirectional relationship is found to exist between diabetes and depression. There is high prevalence of MDD in patients with diabetes, which might be due to the difficulty in adapting to new life style modifications and treatment protocols. On the other hand, depression increases the risk of development of diabetes. The common pathways that link the two diseases include hyperactivation of the hypothalamo–pituitary–adrenal axis with resultant rise in glucocorticoids, peripheral insulin resistance and dysregulation of catecholamine synthesis. Elevated blood glucose levels, inflammation, stress and abnormality in the secretion and signalling pathways of ghrelin and leptin lead to the development of comorbid depression and diabetes^8^. The vascular complications, both microvascular as well as macrovascular complications, and platelet function abnormalities increase several folds in patients with comorbid diabetes and depression. Study by Zahn et al. demonstrated that platelet activation markers CD40, CD62P and soluble CD40L differed significantly in patients having Major Depressive Disorder with and without diabetes^9^. Silić et al. found out a significant difference in the platelet serotonin levels, serum IL-6 and C-Reactive Protein (CRP) levels in patients having MDD with and without metabolic syndrome, which itself is a risk factor for T2DM^10^. Considering the involvement of platelets in mediating the vascular complications associated with these diseases, studies conducted in this area are grossly inadequate. It would be of diagnostic and therapeutic interest to characterise the alterations in platelet activation in patients with comorbid diabetes and Major Depressive Disorder.

The study aimed to quantify and compare these flow cytometry based platelet activation markers between individuals of diabetes with depression, individuals of diabetes without depression and healthy controls and to differentiate the inflammatory state among the three groups.

## Materials and methods

### Study design

This was a hospital based cross-sectional study conducted in the Department of Biochemistry and Non-Communicable Diseases (NCD) Screening Clinic in All India Institute of Medical Sciences (AIIMS), Bhubaneswar. The study followed guidelines enshrined in the Declaration of Helsinki and Tokyo and was duly granted ethical clearance by the Institute Ethical Committee of All India Institute of Medical Sciences (AIIMS) Bhubaneswar (Reg No: ECR/534/Int/OD/2014/RR-17) with the approval number IEC/AIIMS BBSR/PG Thesis/2019-20/19, dated 05th August 2019. The recruitment of subjects, and analysis of the samples were done between August 2019 and June 2021.

A sample size of 114 was estimated, with 38 in each of the three groups, i.e., diabetes with comorbid depression, diabetes without depression and healthy controls, based on the data from previous study by Zahn et al.^9^. Sample size was calculated at a power of 80% and 95% confidence interval to detect an increase of 50% in the percentage of activated platelet cells in subjects with type 2 diabetes mellitus and depression compared to diabetes alone group.

Individuals of both genders within age group 20–60, were included in the study.

The exclusion criteria were:

- antidepressant use for any indications in the last 3 months,
- current insulin therapy,
- major complications of T2DM like retinopathy, nephropathy,
- uncontrolled hypertension,
- major psychiatric disorders (such as schizophrenia, bipolar affective disorder) or substance abuse disorders except tobacco,
- pregnancy,
- suicidal ideation or gestures or psychotic symptoms or severe agitation,
- major organic disorders.

### Study procedure

Thirty-eight individuals (group A) having type 2 diabetes mellitus diagnosed with the American Diabetes Association (ADA) criteria, on stable oral hypoglycaemic drugs for past three months and meeting clinical criteria for Major Depressive Disorder were identified from NCD clinic in AIIMS, Bhubaneswar. Screening and assessment of depression was carried out using Odia version of Patient Health Questionnaire 2 (PHQ2). It is an open access self-reported version of the Primary Care and Evaluation of Mental Disorders (PRIME-MD) by Pfizer Inc. The questionnaire enquires about the frequency of depressed mood and anhedonia for the past two weeks. It is correlated with Diagnostic and Statistical Manual of Mental Disorders-5 (DSM-5). A score of ≥ 3 is indicative of depression^11^. Clinical diagnosis of Major Depressive Disorder was made by a clinical psychologist using DSM-5 criteria.

Hamilton’s Rating Scale for Depression (HAM-D), an open access tool provided by the Mapi Research Trust, was used to assess the severity of depression, which consists of 17 items assessing cognitive, emotional and somatic symptoms of depression. It offers a measure of depression severity with a rating which ranges from 0 to 52. A score of 8–13 is mild depression, 14–18 is moderate depression and more than 18 is severe depression^12^.

Thirty-eight participants (group B) consisting of age and sex matched participants with Type 2 Diabetes mellitus diagnosed with ADA criteria, on stable oral hypoglycaemic drugs for past three months coming to the NCD clinic without signs and symptoms of Major Depressive Disorder were recruited, after getting informed written consent. Equal number of age and sex matched healthy volunteers (group C), i.e., 38 in numbers, without signs and symptoms of Type 2 Diabetes Mellitus or Major Depressive Disorder were recruited as control from AIIMS Bhubaneswar after taking informed written consent.

### Recording of biophysical parameters

The biophysical profile of subjects including age, sex, and relevant history for including / excluding them in the study was taken using a specially designed questionnaire. Height, weight, waist circumference and hip circumference measured using standard techniques. Blood pressure (BP) measured using an automatic BP monitor from the upper arm with hands at heart level in the sitting position after resting the patient for five minutes.

### Blood sampling

Total of 5 ml fasting venous blood was collected from each study subject. Two ml blood sample was collected in an EDTA vacutainer tube for estimation of platelet count and expression of platelet markers assessed by flow cytometry, within four hours of collection. Rest 3 ml was collected in plain vacutainer tube, allowed to stand for 30 min for clot formation and centrifuged at 3500 rotations per minute for 10 min. 500 µl of supernatant serum was stored at − 20 °C in labelled plastic microcentrifuge tubes, which was used for high sensitive C-Reactive Protein (hs-CRP) estimation by immunoturbidimetry and insulin assay by chemiluminescence in batches, details of which are described in the next section.

### Assay of platelet activation markers

The ten colors Navios flow cytometer from Beckman Coulter Ireland, Inc., Lismeehan was used for analysis of the samples. The computer which is attached to the flow cytometer machine stores all the data for each cell, which is then analysed by the inbuilt CXP software.

#### Fluorescent tagged antibodies used in the study

Anti-CD41 antibodies tagged with Electron Coupled Dye (ECD) and Anti-CD42b antibodies tagged with Allophycocyanin (APC) were used as markers for platelet identification. Anti-CD62P antibodies tagged with Phycoerythrin (PE) and Anti-CD63 antibodies tagged with Fluorescein isothiocyanate (FITC) were used as markers for platelet activation. All the four fluorescent tagged antibodies were manufactured by Beckman Coulter Inc., USA.

#### Procedure

**Figure 1:**
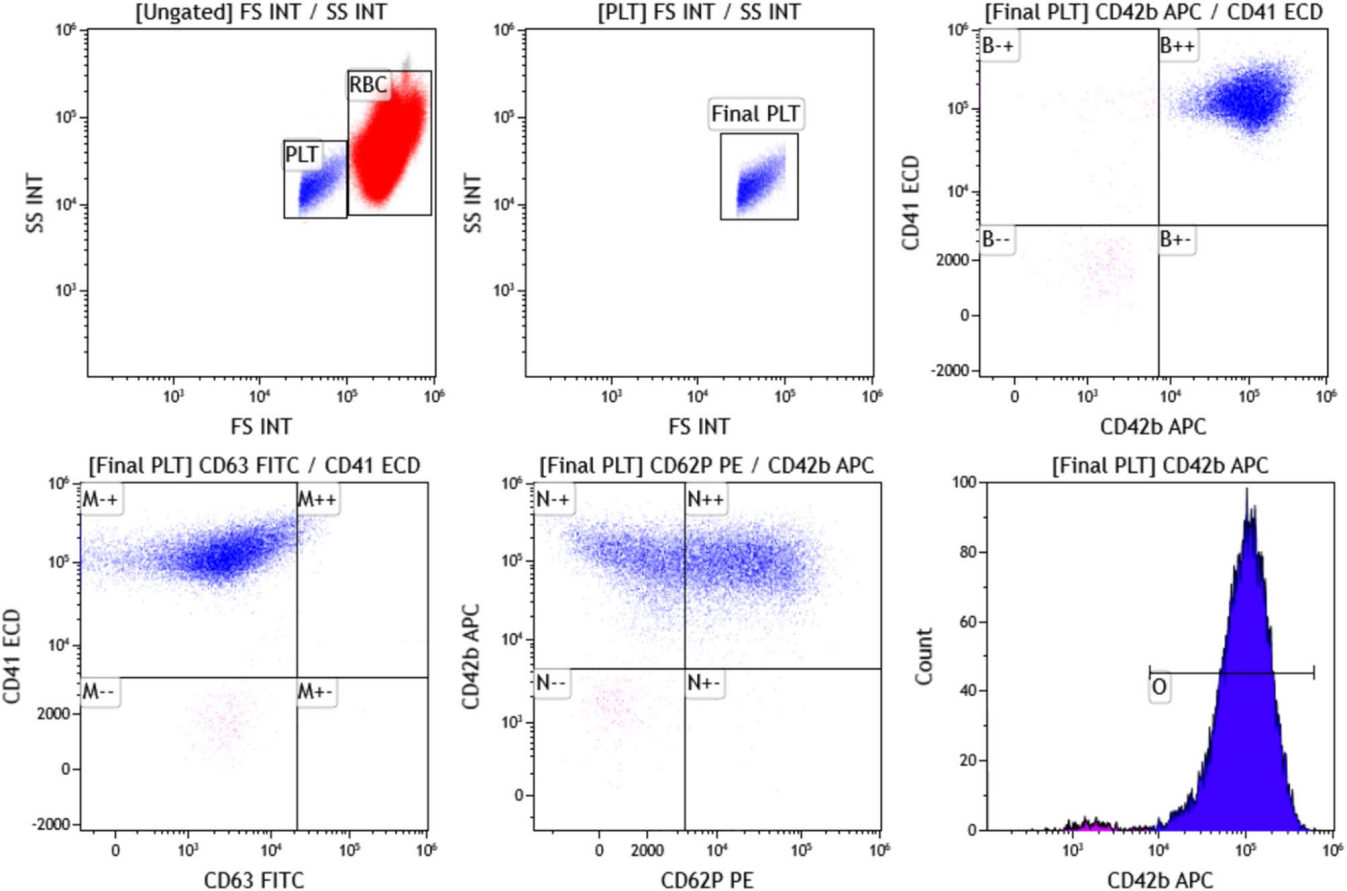
CD62P and CD63 expression on Platelets (PLT), samples were analyzed by flow cytometry, gating by forward/side scatter for platelets.

Flowcheck fluorospheres beads from Beckman Coulter Inc. were run as quality control material before each analysis. The Co-efficient of variation was < 2% for each run. Unstained sample for each of the participant was run before running the prepared sample to rule out autofluorescence. A total of 15,000 platelets were analysed at a flow rate less than 250 platelets/s. We have taken 2 μl of patient’s sample from the EDTA vial (within 4 h of sample collection) in a flow tube and 3 μl of anti-CD41 antibodies tagged with ECD, 2 μl of anti-CD42b antibodies tagged with APC, 5 μl of anti-CD62P antibodies tagged with PE and 10 μl of anti-CD63 antibodies tagged with FITC added. After 15 min of incubation in the dark, 500 μl of Phosphate Buffered Saline (PBS) buffer was added to the flow tube and sample was analysed in the flow cytometer. The gating on platelets was done by forward/side scatter and gating strategy is depicted in Fig. 1. A sample from healthy volunteers (group C) is prepared in the same manner and run along with each case as control.

### Assay of haematological and biochemical parameters

Complete hemogram was performed on Sysmex XT 4000-I fully automated analyser using the anticoagulated 2 ml venous blood sample collected in lavender capped (EDTA) vacutainer in the haematology laboratory of our institute. Fasting plasma glucose (FPG) and post prandial plasma glucose (PPPG) were estimated by hexokinase method, Hemoglobin A_1c_ (HbA_1c_) by turbidimetric immunoinhibition method and hs-CRP by immunoturbidimetry in Beckman Coulter AU 5800 auto analyser. Serum Insulin was estimated by Chemiluminescence Immunoassay (CLIA) in Siemens Advia Centaur XP auto analyser.

### Statistical analysis

Data was analysed using SPSS version 25.0. The skewness of the data was assessed using Shapiro Wilk test. It was found to be not normally distributed. The data was represented in tables as median and interquartile range (IQR). The comparison of medians was done by Kruskal Willis H test. Post hoc analysis was done to compare medians between any two study groups using Dunn Bonferroni test and p value reported after Bonferroni correction. Correlation studies between CD62P and CD63 with other parameters were done using Spearman’s rho correlation study. The p value of < 0.05 was taken significant.

### Ethics approval and consent to participate

The study followed guidelines enshrined in the Declaration of Helsinki and Tokyo and was duly granted ethical clearance by the Institute Ethical Committee of All India Institute of Medical Sciences (AIIMS) Bhubaneswar (Reg No: ECR/534/Int/OD/2014/RR-17) vide their approval number IEC/AIIMS BBSR/PG Thesis/2019-20/19, dated 05th August 2019. All participants read and understood the information leaflet and signed the consent form prior to recruitment.

### Consent for publication

The authors of this manuscript approve this version to be submitted for publication.

## Results

The study has been done to assess the difference in platelet activation markers between individuals of diabetes with depression, individuals of diabetes without depression and healthy controls. Thirty-eight patients with depression were identified. All participants with diabetes and depression in our study were found to have mild depression (HAM-D score of 8–13).

Table 1 compares basic demographic data of the study population. Hundred and fourteen participants with 38 subjects in each group were recruited for the current study. The participants were matched for age and sex. There was no statistically significant difference in the median duration of diabetes among individuals of diabetes with and without depression (p value 0.815). There was no significant difference in BMI or Waist-Hip ratio across the three groups. Systolic as well as diastolic blood pressures varied across the three groups with statistically significant difference in p value. FPG, PPPG and HbA_1c_ levels also varied significantly between the groups.

**Table 1:** Baseline characteristics of the study group.

Parameters	Diabetes with depression (n = 38)	DIABETES without depression (n = 38)	Healthy controls (n = 38)	p value
Age (years)	45.5 (11.3)	45.5 (12)	45 (11.3)	0.996
Males	17 (45%)	17 (45%)	17 (45%)	1.00
Females	21 (55%)	21 (55%)	21 (55%)	1.00
Duration of diabetes (years)	4.5 (3)	4 (3.3)	NA	0.815^a^
Treatment (OHA)	38 (100)	38 (100)	NA	1.00^a^
Number of smokers	5	5	5	1.00
BMI (kg/m^2^)	25.29 (5.24)	25.8 (4.19)	25.48 (2.62)	0.843
Waist/hip ratio	0.90 (0.06)	0.91 (0.02)	0.92 (0.01)	0.308
Systolic BP (mmHg)	120 (9)	124 (4)	114 (4)	< 0.001*
Diastolic BP (mmHg)	80 (14)	82 (6)	78 (8)	0.005*
FPG (mg/dl)	141 (57)	136 (64)	92 (13)	< 0.001*
PPPG (mg/dl)	241.5 (106)	229.5 (104)	119 (13)	< 0.001*
HbA_1c_ (%)	8.28 (3.22)	8.09 (3.49)	5.55 (0.45)	< 0.001*

Results represented as Median (IQR) or Number of subjects (Percentage).

*BP* blood pressure, *OHA* oral hypoglycemic agents, *BMI* body mass index, *FPG* fasting plasma glucose, *PPPG* post prandial plasma glucose, *HbA*_*1c*_ glycated hemoglobin.

p value calculated by Kruskal–Wallis H test for continuous data and chi square test for categorical data.

*p value < 0.05, shows statistically significant difference.

^a^p value compared between participants with diabetes and depression and those without depression, as it was not applicable for healthy controls.

Table 2 compares the platelet activation parameters, hs-CRP levels, insulin levels and Homeostatic Model Assessment for Insulin Resistance (HOMA-IR) scores between the study group. There was significant difference in platelet counts among the groups (p value < 0.001).

**Table 2:** Comparison of platelet parameters, hs-CRP, fasting insulin and HOMA-IR Score between the study groups.

Parameters	Diabetes with depression (n = 38)	Diabetes without depression (n = 38)	Healthy controls (n = 38)	p value
Platelet count (× 10^3^/µl)	322 (95)	315 (79)	250 (56)	< 0.001*
CD62P%	54.00 (20.05)	47.78 (30.73)	27.19 (16.30)	< 0.001*
CD63%	32.99 (18.36)	19.49 (13.13)	15.50 (15.09)	< 0.001*
Hs-CRP (mg/l)	2.43 (3.09)	1.80 (3.94)	1.38 (0.68)	0.018*
Insulin (mIU/l)	10.03 (20.41)	10.53 (18.20)	2.90 (1.0)	< 0.001*
HOMA-IR Score	3.92 (7.91)	3.06 (4.14)	0.70 (0.23)	< 0.001*

Results represented as Median (IQR).

p value calculated by Kruskal–Wallis H test.

*p value < 0.05, shows statistically significant difference.

Post hoc analysis of platelet counts between participants with diabetes and depression and those having diabetes without depression did not differ significantly (Table 3). The percentage of platelets that are positive for CD62P (P-selectin) expression differed significantly between the three groups (p value < 0.001). The median expression of CD62P was more in participants with diabetes and depression compared to those without depression, but it was not statistically significant (p value 0.458). The percentage of platelets that are positive for CD63 expression also differed significantly between the three groups (p value < 0.001). The expression of CD63 was high in patients with diabetes and depression compared to diabetes only group (p value < 0.001). Hs-CRP levels differed significantly across the three groups with a p value of 0.018. Participants with diabetes and depression and those having diabetes without depression did not show significant difference in hs-CRP levels (p value 1.000). There was a significant difference in fasting insulin levels between the three groups (p value < 0.001). Participants with diabetes and depression and those having diabetes without depression did not show significant difference in insulin levels (p value 1.000). The HOMA-IR score also differed significantly between the three groups (p value < 0.001). But there was no significant difference in HOMA-IR score between participants having diabetes with and without depression (p value 1.000).

**Table 3:** Comparison of platelet parameters, hs-CRP, insulin and HOMA-IR score between participants of diabetes with depression and diabetes without depression.

Parameters	Diabetes with depression (n = 38)	Diabetes without depression (n = 38)	p value
Platelet count (× 10^3^/µl)	322 (95)	315 (79)	1.000
CD62P%	54.00 (20.05)	47.78 (30.73)	0.458
CD63%	32.99 (18.36)	19.49 (13.13)	< 0.001*
Hs- CRP (mg/l)	2.43 (3.09)	1.80 (3.94)	1.000
INSULIN (mIU/l)	10.03 (20.41)	10.53 (18.20)	1.000
HOMA-IR score	3.92 (7.91)	3.06 (4.14)	1.000

Results represented as Median (IQR).

Data compared using Dunn–Bonferroni post hoc analysis.

p value adjusted after Bonferroni correction.

*p value < 0.05, shows statistically significant difference.

Table 4 compares the correlation of platelet marker CD62P with hs-CRP, insulin and HOMA-IR. Spearman’s rho correlation coefficient was significant for insulin and HOMA-IR. Table 5 compares the correlation of platelet marker CD63 with hs-CRP, insulin and HOMA-IR. Spearman’s rho correlation co efficient was significant for hs-CRP, insulin and HOMA-IR.

**Table 4:** Correlation of percentage expression of CD62P with hs-CRP, insulin and HOMA- IR score.

Parameters	r value	p value
Hs-CRP (mg/l)	0.096	0.308
INSULIN (mIU/l)	0.335	< 0.001**
HOMA-IR score	0.436	< 0.001**

Data represented as Spearman’s rho Correlation co-efficient ‘r’ with p-value.

*p value significant at level of 0.05.

**p value significant at level of 0.01.

**Table 5:** Correlation of percentage expression of CD63 with hs-CRP, insulin and HOMA- IR score.

Parameters	r value	p value
Hs-CRP (mg/l)	0.204	0.029*
INSULIN (m IU/l)	0.294	0.001**
HOMA-IR	0.363	< 0.001**

Data represented as Spearman’s rho Correlation co-efficient ‘r’ with p-value.

*p value significant at level of 0.05.

**p value significant at level of 0.01.

## Discussion

The study has been done to assess the difference in platelet activation markers between individuals of diabetes with depression, individuals of diabetes without depression and healthy controls.

### Comparison of platelet parameters between the study groups

Platelet count was found to be high in participants with diabetes compared to healthy subjects. This is consistent with the findings of study by Kodiatte et al., Zuberi et al., and Demirtunc et al.^13–15^. The pathogenesis is related to factors associated with vascular disease. Thrombopoietin plays a crucial role in this^16^. Hyperglycemia leads to the synthesis of thrombopoietin in the liver through its action on the Receptor for Advanced Glycation End (RAGE) products. The elevated levels of pro inflammatory markers like IL-6 which have a role in thrombopoiesis adds to the effects of thrombopoietin^17^.

Study by Öztürk et al. compared platelet counts between subjects with moderate depression and healthy controls. There was no significant difference in the two groups. In fact, platelet count was lower in depressed patients which might be due to decreased survival time after activation^18^. Similar was the finding in study by Musselman et al., where basal levels of platelets were slightly higher in healthy controls than depressed patients^19^. The platelet counts of subjects with diabetes and depression differed significantly from healthy controls in the present study mostly due to the effects of diabetes only.

CD62P, a platelet activation marker, is normally found in the alpha granules. They are released upon activation followed by their increased surface expression. CD62P is a C-type lectin, that binds to PSGL1 through the Sialyl Lewis X contributing to inflammation, atherogenesis and thrombogenesis. CD63 is platelet dense granule and lysosomal membrane protein. It is another marker whose surface expression is increased following activation. It plays a role in signal transduction, cell development, activation, vesicle transport and motility, again leading to atherosclerotic changes^20^.

Type 2 Diabetes Mellitus is already a known risk factor for platelet hyperactivation. Israels et al. had established increased expression of both the platelet activation markers CD62P and CD63 in subjects with diabetes compared to healthy subjects^21^. Eibl et al. also identified increased baseline expression of CD62P and CD63 in participants with diabetes^22^.

This study established significant difference in the expression of platelet activation markers between diabetic patients with depression and healthy controls. This is consistent with the study by Morel-Kopp et al. which obtained higher base level expression of the activation markers CD62P and CD63 among participants with depression^23^. Koudouovoh-Tripp et al., in their study established that chronic stress is associated with significantly increased expression of CD63, whereas acute stress raises the expression of both CD62P and CD63^24^. Stress, being an important causative factor for depression, this study also underlines the platelet hyperactivation associated with depression.

Alteration in serotonergic signalling in MDD, along with dysfunction of the hypothalamic–pituitary–adrenal axis and autonomous nervous system are mainly involved in the pathophysiology of platelet hyperactivation in patients with MDD^25^. Platelets themselves cannot synthesize serotonin. But they uptake and store high amounts of peripheral serotonin, produced from sources like the enterochromaffin like cells in the gut. Serotonin executes its action through the serotonergic receptors (5-HT2A) and (5-HT3A) on platelet surfaces. Serotonin receptors and transporters (SERTs) within platelets share great similarity with those of the brain^26^.

The primary objective of the study was to assess whether there was difference in the expression of platelet activation markers between participants with diabetes and depression and those having diabetes without depression. The significant difference in baseline platelet surface expression of CD63 between the two groups underlines the fact that comorbid diabetes and depression can lead to serious vascular complications resulting from platelet hyperactivation. The expression of CD62P was also more in participants with diabetes and depression compared to the diabetes only group, only that it was not statistically significant. Zahn et al. had conducted a study to analyse the expression of platelet activation markers among participants having diabetes with and without depression. They had found that expression of CD62P was significantly different in the two groups. CD63 expression was not included in their study. Instead, another activation marker CD40 was used, which also differed significantly^9^. These studies underline the fact that diabetes and depression not only follow a bidirectional relationship, but also have a combined effect on platelets leading to its hyperactivation.

### Comparison of hs-CRP, insulin and HOMA-IR among the three groups

Our study established higher baseline levels of hs-CRP in patients with comorbid diabetes and depression. Study by Gupta R et al. had already found that hs-CRP was significantly high in participants with diabetes and it correlated positively with HbA_1c_ levels^27^. Study by Pfützner et al. established hs-CRP as a marker for cardiovascular risk in people with T2DM^28^. Major depressive disorder has also been found to be associated with elevated levels of hs-CRP. Researchers observed that median serum levels of hs-CRP increased proportional to the severity of depression^7,29^. Elovainio et al. also identified that higher levels of hs-CRP were associated with increased severity of depression. This rise is attributed to the increased serum levels of the proinflammatory cytokine IL-6^30^. Participants having diabetes with and without depression did not differ significantly in their hs-CRP levels in the current study, which might be due to the fact that all the patients diagnosed with major depressive disorder had, only mild depression. However, Silić et al. in their study, had already identified significant difference in CRP levels in depressed patients with and without metabolic syndrome, which is a risk factor for T2DM^10^. The significant positive correlation of hs-CRP with CD63 elucidates the vicious cycle of diabetes leading to inflammation and vice versa.

There was a significant difference in the insulin levels and HOMA-IR scores between the three study groups, which was expected. Even though no significant difference could be established in insulin levels and HOMA- IR scores among participants with comorbid diabetes and depression compared to those without depression, the role of insulin resistance in the pathogenesis of depression cannot be ignored. Insulin is known to have a neuro modulatory role. This involves the transmission of important neurotransmitters and modulation of the Hypothalamo–Pituitary–Adrenal axis. Hence, brain insulin resistance is a major risk factor for development of depression^31^. Okamura et al. in their study, concluded that patients with depression showed impairment in insulin sensitivity and consequent hyperinsulinemia and these resolved after treatment^32^. Asghar et al. conducted an interventional study and found that insulin sensitivity was related to the severity of depression. They also highlighted the effect of antidepressant treatment in the improvement of insulin sensitivity^33^. Those with high HOMA-IR score and insulin levels, currently not having symptoms of MDD, are still at risk for developing the symptoms. Moreover, insulin resistance can worsen the vascular complications in these patients through mechanisms already explained. This is indicated by the significant positive correlation between the expression of platelet activation markers with insulin levels and HOMA-IR score.

## Conclusion

Patients with comorbid diabetes and depression had significantly high expression of platelet activation markers. They also had a proinflammatory state in their body. This points to the development of a vicious cycle. These patients are at high risk of atherogenesis and related vascular complications. Platelets could be targets of therapeutic interventions for the prevention of such complications in these patients. However, more numbers of prospective studies are needed to establish the causal effect of platelet hyperactivation in patients with comorbid diabetes and depression and their vascular complications.

## Strengths and limitations

To our knowledge, this is the first study from Indian subcontinent to characterize the platelet hyperactivation in patients with comorbid diabetes and depression. Characterization of the proinflammatory state substantiates the pathophysiological mechanisms involved in platelet hyperactivation in participants with comorbid diabetes and depression.

One limitation for this study was that all participants diagnosed with MDD belonged to the category of mild depression. Further studies have to be done to compare the change in expression of the markers with severity of depression.

## Data Availability

Data is available upon request.

## Abbreviations

ADA: American Diabetes Association
AIIMS: All India Institute of Medical Sciences
APC: Allophycocyanin
BDNF: Brain derived natriuretic factor
BMI: Body mass index
CAD: Coronary artery disease
cAMP: Cyclic adenosine monophosphate
CD: Cluster of differentiation
CLIA: Chemiluminescence immunoassay
CRP: C-reactive protein
CVD: Cerebrovascular disease
DSM-5: Diagnostics and statistical manual of mental disorders
ECD: Electron coupled dye
EDTA: Ethylenediaminetetraacetic acid
FITC: Fluorescein isothiocyanate
FPG: Fasting plasma glucose
GTP: Guanosine triphosphate
HAM-D: Hamilton depression rating scale
HbA_1C_: Hemoglobin A_1c_
HDL: High density lipoprotein
HOMA-IR: Homeostatic MODEL ASSESSMENT FOR INSULIN RESISTANCE
5-HT: 5-Hydroxytryptamine
IEC: Institutional Ethics Committee
IL: Interleukin
IQR: Interquartile range
IRS: Insulin receptor substrate
LDL: Low density lipoprotein
MDD: Major depressive disorder
NCD: Non-communicable disease
NO: Nitric oxide
PE: Phycoerythrin
PG: Prostaglandin
PHQ: Patient Health Questionnaire
PKC: Protein kinase C
PPPG: Post prandial plasma glucose
PSGL-1: P-Selectin glycoprotein ligand-1
PVD: Peripheral vascular disease
RAGE: Receptor for advanced glycation end products
ROS: Reactive oxygen species
SERT: Serotonin transporter
T2DM: Type 2 diabetes mellitus
TXB2: Thromboxane B2
VLDL: Very low-density lipoprotein
WHO: World Health Organisation
